# Introgression of Maize Lethal Necrosis Resistance Quantitative Trait Loci Into Susceptible Maize Populations and Validation of the Resistance Under Field Conditions in Naivasha, Kenya

**DOI:** 10.3389/fpls.2021.649308

**Published:** 2021-05-03

**Authors:** Luka A. O. Awata, Beatrice E. Ifie, Eric Danquah, MacDonald Bright Jumbo, L. Mahabaleswara Suresh, Manje Gowda, Philip W. Marchelo-Dragga, Michael Scott Olsen, Oluwaseyi Shorinola, Nasser Kouadio Yao, Prasanna M. Boddupalli, Pangirayi B. Tongoona

**Affiliations:** ^1^Directorate of Research, Ministry of Agriculture and Food Security, Juba, South Sudan; ^2^West Africa Centre for Crop Improvement (WACCI), College of Basic and Applied Sciences, University of Ghana, Legon, Ghana; ^3^International Crops Research Institute for the Semi-Arid Tropics (ICRISAT), Bulawayo, Zimbabwe; ^4^International Maize and Wheat Improvement Center (CIMMYT), World Agroforestry Centre (ICRAF), Nairobi, Kenya; ^5^Department of Agricultural Sciences, College of Natural Resources and Environmental Studies, University of Juba, Juba, South Sudan; ^6^Biosciences eastern and central Africa (BecA) Hub, International Livestock Research Institute (ILRI), Nairobi, Kenya; ^7^John Innes Centre, Norwich, United Kingdom

**Keywords:** maize, backcross, kompetitive allele specific PCR, alleles, maize lethal necrosis, introgression

## Abstract

Maize lethal necrosis (MLN), resulting from co-infection by maize chlorotic mottle virus (MCMV) and sugarcane mosaic virus (SCMV) can cause up to 100% yield losses in maize in Africa under serious disease conditions. Maize improvement through conventional backcross (BC) takes many generations but can significantly be shortened when molecular tools are utilized in the breeding process. We used a donor parent (KS23-6) to transfer quantitative trait loci (QTL) for resistance to MLN into nine adapted but MLN susceptible lines. Nurseries were established in Kiboko, Kenya during 2015–2017 seasons and BC_3_F_2_ progeny were developed using marker assisted backcrossing (MABC) approach. Six single nucleotide polymorphism (SNP) markers linked to QTL for resistance to MLN were used to genotype 2,400 BC_3_F_2_ lines using Kompetitive Allele Specific PCR (KASP) platform. We detected that two of the six QTL had major effects for resistance to MLN under artificial inoculation field conditions in 56 candidate BC_3_F_2_ lines. To confirm whether these two QTL are reproducible under different field conditions, the 56 BC_3_F_2_ lines including their parents were evaluated in replicated trials for two seasons under artificial MLN inoculations in Naivasha, Kenya in 2018. Strong association of genotype with phenotype was detected. Consequently, 19 superior BC_3_F_2_ lines with favorable alleles and showing improved levels of resistance to MLN under artificial field inoculation were identified. These elite lines represent superior genetic resources for improvement of maize hybrids for resistance to MLN. However, 20 BC_3_F_2_ lines were fixed for both KASP markers but were susceptible to MLN under field conditions, which could suggest weak linkage between the KASP markers and target genes. The validated two major QTL can be utilized to speed up the breeding process but additional loci need to be identified between the KASP markers and the resistance genes to strengthen the linkage.

## Introduction

Maize is the major food crop in Sub-Saharan Africa, however, its productivity remains low due to various production constraints such as biotic and abiotic factors. Recently, maize lethal necrosis (MLN) has emerged as one of the most deadly maize diseases in the region with high yield losses. MLN is caused by co-infection of maize plants by Maize chlorotic mottle virus (MCMV) in combination with any of the cereal viruses in the family *Potyviridae*, such as Sugarcane mosaic virus (SCMV), Maize dwarf mosaic virus (MDMV), or Wheat streak mosaic virus (WSMV). In east Africa, it has been established that it is mostly SCMV in combination with MCMV causing MLN. MLN disease causing viruses are transmitted by vectors such as thrips and beetles for MCMV (Nault et al., 1978), and aphids for potyviruses such as SCMV and MDMV (Brandes, 1920; Pemberton and Charpentier, 1969). MLN can cause losses in maize ranging from 30 to 100% depending on disease pressure (Gowda et al., 2018; Awata et al., 2019). Elite maize lines used in countries like South Sudan and others are highly susceptible to MLN and farmers risk losing their crops and money if MLN is not controlled. Therefore, efforts to develop high yielding varieties with resistance to MLN are urgently required. Breeding for host resistance can provide added advantage to farmers in terms of costs as compared to spraying against the vectors using chemicals, which is expensive and results in pollution to the environment. Conventional backcrossing is a routine breeding approach used for introgression of novel genes into the genetic backgrounds of adapted but susceptible germplasm but requires 8–10 generations to develop lines with desired characteristics. Studies to identify genomic regions associated with MLN resistance using linkage mapping revealed three major quantitative trait loci (QTL) on chromosomes 3, 6, and 9 that were consistently detected in at least two populations (Gowda et al., 2018) with recessive genetic effects. Introgression of genes for MLN resistance into the adapted lines using molecular markers is a quick option for fast-tracking development of varieties with resistance to MLN. Marker assisted backcrossing (MABC) has been widely used in improvement of maize for traits of economic importance including resistance to diseases (Lübberstedt et al., 2006; Muthusamy et al., 2014; Feng et al., 2015; Rasheed et al., 2016). Therefore, use of MABC can be used to speed up identification of fixed QTL conferring resistance to MLN into the backgrounds of adapted but susceptible maize lines. Kompetitive allele specific PCR (KASP) markers, developed by LGC Genomics (Teddington, United Kingdom),^1^ is a PCR-based homogeneous fluorescent single nucleotide polymorphism (SNP) genotyping system. It has the power to detect single nucleotide polymorphism at a specific locus using dual Fluorescent Resonance Energy Transfer (FRET; Semagn et al., 2014). KASP has high throughput, low cost, and more roboust than other genotyping assays such as Restriction Fragment Length Polymorphism (RFLP), Randomly Amplified Polymorphic DNA (RAPD), and Amplified Fragment Length Polymorphism (AFLP), which require longer time and have higher cost per sample. KASP technology has been utilized on various crops including wheat (Rasheed et al., 2016) and cordgrass (Graves et al., 2016). The objectives of this study were: (i) introgression of MLN resistance from a resistant source into adapted but susceptible elite maize lines using the KASP method; (ii) validate the effect of the introgressed QTL for resistance to MLN in lines evaluated under MLN artificial inoculation in the field; and (iii) identify resistant lines for future breeding.

## Materials and Methods

### Genetic Materials

Genetic materials were provided by CIMMYT Global Maize Program and consisted of two maize categories: (i) MLN resistant donor line (KS23-6) developed by Kasetsart University in Thailand, which is a yellow maize line and considered suitable parent for maize improvement in Africa because of its tropical adaptation; (ii) 19 elite but MLN susceptible white CIMMYT lines, with diverse tropical backgrounds and each line belonging to one of the two heterotic groups (A and B) and are commonly used for hybrid development in the region including South Sudan due to their high yield performance and resistance to major foliar diseases (Supplementary Appendix 1).

### Development of Bi-Parental Backcross Populations

Crossing blocks to develop backcross (BC) populations were established at CIMMYT in Kiboko, between July 2015 and 2017 cropping seasons. Kiboko is located within the dry-mid altitude environment at 37^0^ 75'E and 20 15'S, and 975 masl in Makueni County, Kenya, with mean temperature ranging from 14.3 to 35.1°C (Ziyomo and Bernardo, 2013; Odiyo et al., 2014). The first crossing block was set up in April 2015 and bi-parental populations were formed by crossing KS23-6 as pollen donor to the 19 selected elite but MLN susceptible lines (Awata et al., 2018). Adequate moisture was supplied through drip irrigation and standard agronomic practices and nursery management were applied. A nursery to develop backcross populations was established in October 2015, where 19 F_1_ populations were grown in single-row plots of 4.0 m spaced at 0.75 × 0.25 m (Table 1). Larger population size was required so that both major QTL associated with resistance to MLN could be detected (Bouchez et al., 2002; Hospital, 2003; Vales et al., 2005; Ribaut and Ragot, 2007). Therefore, 10 F_1_ individual plants (females) were tagged per population and each backcrossed to its recurrent parent (males), hence BC_1_F_1_ populations were developed (Brown and Caligari, 2008; Acquaah, 2012). The BC_1_F_1_ progeny were planted in March 2016. About 60 agronomically healthy BC_1_F_1_ individuals (females) were labeled within each population and backcrossed to their respective recurrent parents (males), to generate BC_2_F_1_. The BC_2_F_1_ populations were planted in the nursery in August 2016 and about 60 clean plants in each population were tagged and backcrossed to the recurrent parents where BC_3_F_1_ populations were generated. The BC_3_F_1_ populations were planted in January 2017 and tissue samples were collected from this nursery and sent to LGC in United Kingdom and genotyped with 100 markers for MLN and 250–300 individuals per population were selected based on the marker information received from LGC. The selected 250–300 individuals for each population were manually self-pollinated and BC_3_F_2_ seeds obtained (Table 2). The BC_3_F_2_ populations were eventually used for genotyping during the subsequent seasons and for trials in Naivasha during field evaluation under MLN artificial inoculation.

**Table 1:** List of 19 bi-parental crosses generated and used to develop BC_3_F_2_ lines genotyped for resistance to MLN using two polymorphic SNP markers linked to major QTL for resistance to MLN in BecA-ILRI Hub Lab in July 2017.

SN	Population	Bi-parental cross	SN	Population	Biparental cross
1	Pop1	KS23-6 × CML567	11	Pop11	KS23-6 × CML547
2	Pop2	KS23-6 × CML568	12	Pop12	KS23-6 × CML566
3	Pop3	KS23-6 × CML442	13	Pop13	KS23-6 × CML569
4	Pop4	KS23-6 × CML537	14	Pop14	KS23-6 × CML570
5	Pop5	KS23-6 × CML548	15	Pop15	KS23-6 × CKL05017
6	Pop6	KS23-6 × CML572	16	Pop16	KS23-6 × CKL05019
7	Pop7	KS23-6 × CKDHL0106	17	Pop17	KS23-6 × CML539
8	Pop8	KS23-6 × CKDHL0323	18	Pop18	KS23-6 × CML540
9	Pop9	KS23-6 × CML444	19	Pop19	KS23-6 × CKDHL0186
10	Pop10	KS23-6 × CML511	20	KS23-6^*^	

* Donor parent for resistance to MLN.

**Table 2:** List of selected BC_3_F_2_ lines including resistant and susceptible parents evaluated for two seasons under artificial inoculations for resistance to MLN in Naivasha in 2018.

SN	Pedigree	SN	Pedigree
1	(CKDHL0106 * 2/KS523-5):B-1110 > 1,016	32	(CML511 * 2/KS23-6):B-1154 > 1,037
2	(CKDHL0186 * 2/KS23-6):B-1019 > 1,033	33	(CML511 * 2/KS23-6):B-1154 > 1,037
3	(CKDHL0186 * 2/KS23-6):B-1019 > 1,033	34	(CML511 * 2/KS23-6):B-1154 > 1,037
4	(CKDHL0106 * 2/KS523-5):B-1110 > 1,040	35	(CML511 * 2/KS23-6):B-1083 > 1,008
5	(CKDHL0106 * 2/KS523-5):B-1110 > 1,040	36	(CML511 * 2/KS23-6):B-1154 > 1,037
6	(CKDHL0106 * 2/KS523-5):B-1110 > 1,040	37	(CML511 * 2/KS23-6):B-1083 > 1,008
7	(CKDHL0106 * 2/KS523-5):B-1110 > 1,016	38	(CML511 * 2/KS23-6):B-1083 > 1,008
8	(CKDHL0106 * 2/KS523-5):B-1110 > 1,016	39	(CML511 * 2/KS23-6):B-1154 > 1,037
9	CKDHL0106	40	CML511
10	(CKDHL0106 * 2/KS523-5):B-1110 > 1,016	41	(CML511 * 2/KS23-6):B-1154 > 1,037
11	CML444	42	(CML547 * 2/KS23-6):B-1092 > 1,019
12	(CML444 * 2/KS23-6):B-1118 > 1,008	43	(CML547 * 2/KS23-6):B-1092 > 1,019
13	(CML444 * 2/KS23-6):B-1118 > 1,008	44	(CML547 * 2/KS23-6):B-1092 > 1,019
14	(CML444 * 2/KS23-6):B-1118 > 1,008	45	(CML547 * 2/KS23-6):B-1028 > 1,008
15	(CML511 * 2/KS23-6):B-1154 > 1,037	46	(CML547 * 2/KS23-6):B-1092 > 1,019
16	(CML511 * 2/KS23-6):B-1083 > 1,008	47	(CML547 * 2/KS23-6):B-1028 > 1,008
17	(CML511 * 2/KS23-6):B-1083 > 1,008	48	(CML547 * 2/KS23-6):B-1028 > 1,008
19	(CML511 * 2/KS23-6):B-1154 > 1,037	49	(CML547 * 2/KS23-6):B-1092 > 1,019
20	(CML511 * 2/KS23-6):B-1154 > 1,041	50	(CML547 * 2/KS23-6):B-1028 > 1,008
21	(CML511 * 2/KS23-6):B-1083 > 1,008	51	(CML547 * 2/KS23-6):B-1028 > 1,008
22	(CML511 * 2/KS23-6):B-1154 > 1,037	52	(CML547 * 2/KS23-6):B-1028 > 1,008
23	(CML511 * 2/KS23-6):B-1083 > 1,008	53	(CML547 * 2/KS23-6):B-1028 > 1,008
24	(CML511 * 2/KS23-6):B-1154 > 1,037	54	(CML547 * 2/KS23-6):B-1092 > 1,019
25	(CML511 * 2/KS23-6):B-1154 > 1,037	55	(CML547 * 2/KS23-6):B-1028 > 1,008
26	(CML511 * 2/KS23-6):B-1083 > 1,008	56	KS23-6
27	(CML511 * 2/KS23-6):B-1154 > 1,037	57	(CML547 * 2/KS23-6):B-1092 > 1,019
28	(CML511 * 2/KS23-6):B-1083 > 1,008	58	CML547
29	(CML511 * 2/KS23-6):B-1083 > 1,008	59	(CML547 * 2/KS23-6):B-1028 > 1,008
30	KS23-6	60	(CML547 * 2/KS23-6):B-1028 > 1,008
31	(CML511 * 2/KS23-6):B-1083 > 1,008	61	(CML547 * 2/KS23-6):B-1092 > 1,019
31	(CML511 * 2/KS23-6):B-1083 > 1,008	62	(CML547 * 2/KS23-6):B-1028 > 1,008

### Genotyping and Marker-Trait Association Analysis

In the present study, we used 19 SNP markers linked to two major QTL associated with resistance to MLN on chromosomes 3 and 6 developed by CIMMYT from two association mapping (AM) panels of diverse tropical/subtropical maize lines (Supplementary Table S1). The two markers explained 33.8% of the phenotypic variance for MLN resistance in the two panels (Gowda et al., 2015). These SNPs were used to design 21 KASP primers for resistance to MLN at BecA-ILRI Hub Laboratory, with three primers for each SNP; two allele-specific forward primers (5'-3') and one common reverse primer (3'-5'; Supplementary Appendix 2).

The 20 parent lines were planted in tray pots filled with sterile soil in a screen house in July 2017. Two seeds were sown per pot with four replicates and each pot was considered an entry so that a total of 80 entries were generated. No fertilizer was applied and soil moisture was maintained using irrigation. Leaf samples were collected from each entry 14 days after seedling emergence. Trays were carried aseptically onto the bench in the laboratory to avoid contamination. About 8–10 leaf disks per plant were collected from tips of the youngest leaves of each entry using a leaf puncher and placed in a 1.2 ml Eppendorf tube (F and S Scientific Ltd., Kenya) arranged in a 96-well plate placed in an ice bucket. At least 94 wells were filled with samples, while two more were filled with no treatment control (NTC) using ddH_2_0.

DNA extraction procedures were based on Cetyl Trimethyl Ammonium Bromide (CTAB) protocols developed by BecA-ILRI Hub Laboratory with some modifications. After sampling, leaf samples were frozen in liquid nitrogen for 2–5 min and ground into fine powder using Geno/Grinder (SPEXSamplePrep^(R)^ 2000, 2 Dalston Gardens Stanmore, HA7 1BQ, United Kingdom). Genomic DNA was extracted from the fine powder samples following the CTAB protocols. DNA concentration was measured using a spectrophotometer (NanoDrop 8000 Spectrophotometer, Thermo Fisher Scientific, Wilmington, DE, United States) and was adjusted to 50 ng/μl using Nuclease-free water (Patterson et al., 2017). DNA samples of poor quality were discarded and therefore, only samples with high DNA quality were retained and used in this study. Extracted genomic DNA samples were clearly labeled and stored at −20°C until further use (Kusza et al., 2018). The 21 primers generated above were screened for polymorphism to MLN resistance using the 20 parent lines (one resistant donor and 19 susceptible lines) described earlier. Genotypic analyses were conducted using KASP platform established in BecA-ILRI Hub Laboratory in Nairobi, Kenya.

Kompetitive allele specific PCR assays refer to a combination of three SNP-specific primers (two forward and one common reverse), while master mix contained FRET cassettes, free nucleotides, and enzyme components. These materials are required for running the PCR therefore, they should be secured before initiating any PCR process. Correct combination of assays and master mix is vital for obtaining good PCR output and KASP results. In the present study, both SNP specific KASP Assays and KASP master mix (2xKASP) were ordered from LGC Genomics (LGC Group, Queens Road, Teddington, Middlesex, TW11 0LY, United Kingdom; see Footnote 1^1^). Each assay was supplied in a single 2D barcoded tube with the allele-specific forward primers differing in their tail sequences: allele-1 tail was labeled with fluorescein amide (FAM) oligo-sequence; allele-2 tail contained Hexachloro-flourescein (HEX) oligo-sequence (Rasheed et al., 2016; Patil et al., 2017; Patterson et al., 2017). KASP master mix was composed of universal FRET cassette dyes (FAM and HEX), ROX™ passive reference dye, KASPTaq™ DNA polymerase, free nucleotides, and MgCl_2_ in an optimized buffer solution. On arrival, the assays and the mix were stored at −20°C until further use. The SNP-specific KASP assays (primers) and universal KASP master mix (2xPCR) obtained above were used to constitute KASP reaction mix. Universal KASP master mix was readily obtained from the supplier as described earlier. The reaction mix was constituted in a 100 μl volume as follows:

**Table:** 

FAM (forward primer)	=	12 μl
HEX (forward primer)	=	12 μl
Common primer	=	30 μl
Water (ddH_2_0)	=	46 μl
Total	=	100 μl

Volume of each of KASP master mix and Assay mix required for KASP reaction was aliquoted into new 1.5 ml Eppendorf tube using a pipette as follow:

**Table:** 

KASP master mix	=	2.5 μl × N × 1.5
Assay mix	=	0.07 μl × N × 1.5

where *N* = number of wells to be filled per reaction, 1.5 = error factor. The KASP master mix and Assay mix were then combined into a common volume to constitute a KASP genotyping reaction mix (cocktail). A volume of KASP genotyping reaction mix was aliquoted in each reaction well of a four-quadrant 384-well plate (one quadrant = 96 wells) as below:

**Table:** 

KASP reaction mix	=	2.5 μl
DNA sample	=	2.5 μl
Total KASP reaction mix per well	=	5.0 μl

**Figure 1:**
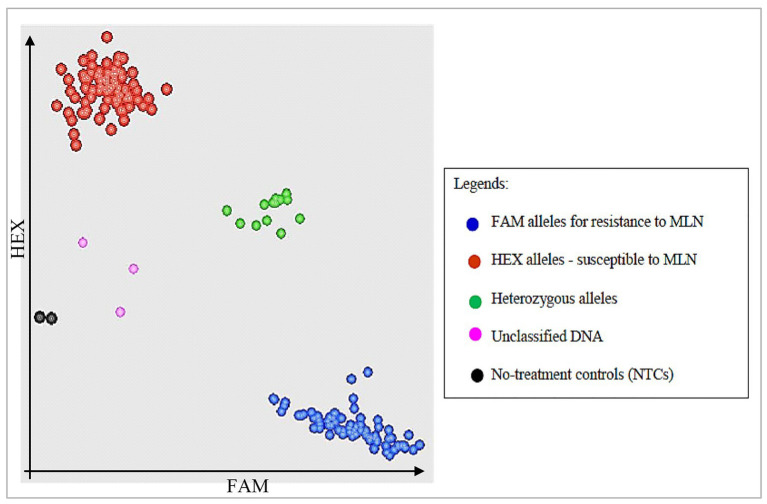
Schematic illustration of kompetitive allele specific PCR (KASP) cluster plots showing segregation of bi-parental backcross (BC) populations for alleles for resistance and susceptibility to maize lethal necrosis (MLN). Fluorescein amide (FAM) alleles for resistance to MLN are clustered on x-axis, while susceptible Hexachloro-flourescein (HEX) alleles are on y-axis.

Assays were put in wells in a 96-well plate where two last wells were stamped with sterile water as NTCs. KASP reaction plate was sealed with optically clear seal and centrifuged at 3,500 rpm for 10–15 s. The template was then amplified using KASP thermal cycling reaction in a FRET-capable plate reader (qPCR) instrument (GeneAmp® PCR System 9700, Roche Molecular Systems, Inc., United States). Unlike other traditional thermal cyclers where three thermal regimes are required, only two temperature steps were used for KASP thermal cycling. In the first step, KASP activation and DNA denaturation was completed in one cycle and at higher temperature (94°C) in 15 min. Step 2 involved two cycle regimes: Annealing and elongation completed in 10 cycles and at lower temperatures of 61–55°C in 60 s; and the last step required 26 cycles where DNA denaturation occurred at 94°C in 20 s; while annealing and elongation occurred at 55°C in 60 s. Sample amplifications were performed for 30, 35, and 40 cycles. Running the PCR for more than two cycles was necessary to provide an opportunity to select a cycle with the best and clearest clustering of samples for further genotyping (Rasheed et al., 2016; Patil et al., 2017). After KASP reactions were complete, plates were read using fluorescence plate reader BMG FLUOstar Omega software (LGC, Queens Road, Teddington, Middlesex, TW11 0LY, United Kingdom). Data was then displayed as cluster plots where FAM values were plotted on the x-axis, HEX values plotted and on y-axis and heterozygous values clustered on the diagonal (Figure 1). KASP reactions with NTCs were plotted at the origin (represented by black dots) since they did not generate any fluorescence. Each data point on the cluster plot represented the fluorescence signal of individual DNA samples. Based on the plate readings, two SNP markers (S3_146250249 and S3_146363360) associated with major QTL for resistance to MLN (Table 3) showed polymorphisms in the parents, hence were used for genotyping of the backcross populations.

Seeds of BC_3_F_2_ progeny obtained from the nine populations were planted in a nursery in Kiboko in July 2017. Leaf samples were collected from 250 to 300 healthy individual BC_3_F_2_ plants per population 15–20 days after seedling emergence. The leaf sampling techniques followed the procedures adopted by BecA-ILRI Hub Laboratory for field sample collection. Each 96-well plate was labeled and only 94 wells were filled with samples, while two wells were the NTCs as described earlier. Tubes were securely closed with perforated strip caps. To enhance moisture reduction and drying of the leaf samples, a 50 g bag of silica gel (Grade 4) obtained from BecA-ILRI Hub Laboratory was put on top of each 8 × 12 strip tubes in a plate, covered and securely tied with a rubber band, then packaged in a zip-tight polythene bag. Samples were transported to BecA-ILRI Laboratory in Nairobi within 12–24 h and stored at room temperature on the bench until DNA extraction was initiated. Genomic DNA extraction and genotyping followed the same protocol as described for parental screening. The two SNP markers identified above were used for the genotyping using Omega software. Fifty-seven BC_3_F_2_ progeny carrying the two markers were identified through the genotyping.

Data from Omega software were imported into KlusterCaller software (LGC Genomics, Queens Road, Teddington, Middlesex, TW11 0LY, United Kingdom) and cluster plots were normalized using ROX (passive reference dye) then called into X:X, X:Y, and Y:Y alleles depending on the corresponding genotype. Results were then exported onto Excel 2016 version, following 96-well plate format and calls were converted into specific alleles where X:X represented homozygous alleles for FAM, Y:Y represented homozygous alleles for HEX, and X:Y represented segregating (heterozygous) alleles (FAM/HEX; Graves et al., 2016; Kusza et al., 2018). The KASP analysis revealed that six of the SNP primers were polymorphic and could clearly discriminate between resistance alleles of donor parent (KS23-6) and the susceptibility alleles of the recipient parents (Table 4). The remaining 15 SNPs were monomorphic and could not differentiate between the resistant and susceptible parents. Previous reports confirm that two out of the six polymorphic SNPs are linked to major QTLs for resistance to MLN, while the remaining four markers are also for resistance but with minor effects (Gowda et al., 2015, 2018). As a result, only the two major SNPs (S3_146250249 and S3_146363360) were retained and used as markers for genotyping of the BC_3_F_2_ populations.

**Table 4:** List of six polymorphic KAPS primers validated for resistance to MLN using 20 parent lines including a resistant donor parent.

Assay code	Primer name	Primer sequence	Remarks
B0051_FAm	S3_44062810_FAm	gaaggtgaccaagttcatgctATCCGCCTTATTGCCGGg	
B0051_HEX	S3_44062810_HEX	gaaggtcggagtcaacggattATCCGCCTTATTGCCGGa	Polymorphic, linked to QTL with minor effects
B0051_COm	S3_44062810_COm	AGGATTAACGACGGGAAGGT	
B0054_FAm	S3_146966676_FAm	gaaggtgaccaagttcatgctGTCCTGCTGCTGGAGCGt	
B0054_HEX	S3_146966676_HEX	gaaggtcggagtcaacggattGTCCTGCTGCTGGAGCGc	Polymorphic, linked to QTL with minor effects
B0054_COm	S3_146966676_COm	GTAGGCGTCCCGGATGAT	
B0056_FAm	S3_146250249_FAm	gaaggtgaccaagttcatgctCTACCCATCCGCCTGCTt	
B0056_HEX	S3_146250249_HEX	gaaggtcggagtcaacggattCTACCCATCCGCCTGCTg	Polymorphic, linked to QTL with major effects
B0056_COm	S3_146250249_COm	CACCTGGCACGGAGAGAAG	
B0057_FAm	S3_146363360_FAm	gaaggtgaccaagttcatgctACCAGGACAGGTATCTAACGCc	
B0057_HEX	S3_146363360_HEX	gaaggtcggagtcaacggattACCAGGACAGGTATCTAACGCt	Polymorphic, linked to QTL with major effects
B0057_COm	S3_146363360_COm	CGTACCAGGTCTGAGCACAA	
B0060_FAm	S6_21007530_FAm	gaaggtgaccaagttcatgctGCAAAAATCACAGCCGATCg	
B0060_HEX	S6_21007530_HEX	gaaggtcggagtcaacggattGCAAAAATCACAGCCGATCa	Polymorphic, linked to QTL with minor effects
B0060_COm	S6_21007530_COm	CCGGGCCTAAAGCCTAATAC	
B0065_FAm	S6_157568432_FAm	gaaggtgaccaagttcatgctGCATAGAAATAAAATGAGACAAGGg	
B0065_HEX	S6_157568432_HEX	gaaggtcggagtcaacggattGCATAGAAATAAAATGAGACAAGGt	Polymorphic, linked to QTL with minor effects
B0065_COm	S6_157568432_COm	ATCCATGTTGTCCCTCCGTA	

### Phenotypic Evaluation of Backcross Populations

The 57 BC_3_F_2_ lines selected from the molecular analysis for this study, including four checks (one MLN resistant and three MLN susceptible) and their parents were evaluated for two seasons in 2018 to validate the effects of the two markers for resistance to MLN under MLN artificial inoculation in the field. The two markers, located approximately 0.62 cM (113111 nt) apart on chromosome 3, were putatively identified in different populations using genome-wide association studies (Gowda et al., 2015). Experiments were conducted in Naivasha, Kenya (36^°^26E; 0°43S; 1896 masl; 677 mm rainfall; and 24.9°C; Ziyomo and Bernardo, 2013; Odiyo et al., 2014). To reduce soil borne diseases and pest infections, seeds were treated with Apron Star WS seed treatment chemical at the rate of 20 g/kg of seed. Recommended fertilizer rates were adopted and applied in two separate regimes (Izge and Dugje, 2011). The trial was laid out in an alpha lattice design with two replications, and a one-row plot of 3.0 m, with spacing of 0.75 m between rows and 0.3 m between plants. Two seeds were planted per hill and thinned to one plant per hill 3 weeks post emergence, resulting in a total of 10 plants per row. Standard agronomic practices were maintained (Gowda et al., 2015; Mahuku et al., 2015).

### Artificial MLN Inoculation

Infected leaf samples collected from the field were cut into small pieces and ground using a mortar and pestle in a grinding buffer of 1:10 dilution ratio (10 ml potassium-phosphate, pH 7.0) as described by Gowda et al. (2015) and Mahuku et al. (2015). The resulting sap extract was centrifuged for 2 min at 12,000 rpm. Celite powder was added to the decanted sap extract at the rate of 0.02 g/ml. A susceptible hybrid was inoculated by rubbing sap extract onto the leaves at the two leaf stage and infected maize plants grown in separate, sealed greenhouses that were maintained for each of SCMV and MCMV inoculum production. Three weeks before inoculation of the experimental materials, ELISA test was conducted on random samples of leaves from the plants infected with SCMV and MCMV, respectively, to confirm presence and purity of the inoculum (Gowda et al., 2018). Separate extracts from the SCMV and MCMV infected plants were prepared at the ratio of one part of leaf sample: 20 parts of phosphate buffer. The two extracts were then mixed to form MLN inoculum at the ratio of four parts of SCMV: one part MCMV (weight/weight; Gowda et al., 2015). In order to keep uniform disease pressure, plants were inoculated using a motorized, backpack mist blower (Solo 423 mist Blower, 12 L capacity) with an open nozzle (2-in diameter) delivering inoculum spray at a pressure of 10 kg/cm^2^ (Gowda et al., 2015). Two inoculations were applied at 4th and 5th week after planting (Gowda et al., 2018). Spreader rows of susceptible maize hybrid (H614) were also planted as border rows along the experiment to enhance disease spread and intensity (Tivoli et al., 2006). Nitrogen (Urea) and Phosphorus (DAP) fertilizers were applied as described by Makumbi et al. (2015). Drip irrigation was used to provide moisture and all other agronomic practices relating to maize production were followed according to CIMMYT procedures for field practices.

A quantitative scale of 1–9 introduced by Reddy and Singh (1984) was used for recording data on MLN severity, where 1 = resistant (no symptoms); 2 = resistant to moderately resistant (isolated plants with very few lesions in the lower canopy); 3 = moderately resistant (1–5 leaves with symptoms in the lower canopy); 4 = moderately resistant to moderate (most or all plants with one or more leaves affected in the lower canopy); 5 = moderate (most or all plants with many leaves affected on plant, few leaves affected in the mid canopy); 6 = moderate to moderately susceptible (numerous lesions on most leaves in the mid canopy, limited defoliation in lower canopy); 7 = moderately susceptible (same as six, but limited defoliation in mid canopy and severe defoliation in lower canopy); 8 = moderately susceptible to susceptible (severe defoliation in mid canopy and limited defoliation in upper canopy); and 9 = susceptible (complete plant necrosis; Ngugi et al., 2002; Meyer and Pataky, 2010). The scale of 1–9 was considered more convenient (in terms of recording and time) compared to 1–5 because: 1 = 1; 1.5 = 2; 2 = 3; 2.5 = 4; 3 = 5; 3.5 = 6; 4 = 7; 4.5 = 8; and 5 = 9. Disease severity was recorded four times, beginning 21 days from the date of first inoculation (Mahuku et al., 2015; Mezzalama, 2015; Gowda et al., 2018).

Disease severity data were first tested for independence, normal distribution and constant variance (GenStat ver 12.0). ANOVA was performed using the restricted maximum likelihood (REML) model established in SAS 9.4 (SAS institute Inc, 2016), based on lattice incomplete block analysis as follows:

$$Y_{ijk}=\mu +g_{i}+r_{j}+b_{kj}+e_{ijk}$$

where *Y_ijk_* is the disease severity of the ith genotype in the jth replication of the kth incomplete block, *μ* is the population mean, *g_i_* is the genetic effect of the ith genotype, *r_j_* is the effect of the jth replication, *b_kj_* is the effect of the kth incomplete block in the jth replication, and *e_ijk_* is the error term. Genotype was considered fixed, while season, replication, and block within replication were considered random. Best linear unbiased prediction estimates (BLUP) for the populations were generated and used for further QTL analysis in the present study.

#### Marker-Trait Association Analysis

The BLUP estimate for phenotypic data was generated for each genotype as described above. Co-segregation of loci with phenotype was detected by comparing allele type with the phenotype. Positive co-segregation was declared when a genotype showed resistance allele and MLN score of below 4.0 (in the scale of 1–9). Relationship between allele and phenotype was confirmed by splitting the BC_3_F_2_ progeny into resistant and susceptible groups and means of the two groups compared using a *t*-test. Further, significance of differences between variances of means was determined using *F*-tests.

## Results

### Screening of Parental Lines

A total of 21 KASP primers designed from 19 SNP markers, originally developed by CIMMYT Global Maize Program, were tested for polymorphism to MLN resistance using 20 parental lines that were involved in development of bi-parental backcross populations. Six KASP markers showed polymorphism for resistance to MLN among 9 of the 19 bi-parental backcross populations used. As a result, the remaining 11 populations were eliminated. Two of the six polymorphic KASP markers (*S3_146250249* and *S3_146363360*) were previously reported to be linked to major QTL associated with resistance to MLN (Gowda et al., 2015). Therefore, they were retained for this study. The preliminary KASP analysis revealed that recurrent parents of the nine selected populations were fixed for susceptibility alleles, while the donor parent was homozygous for resistance alleles for both markers (Table 3).

**Table 3:** Segregation of nine recurrent parents and a donor parent genotyped for resistance to MLN using two SNP markers.

SN	Parent	SNP1: Chr3_146250249 (T/G)	Chr3_146363360 (C/T)
1	CML548	G:G	T:T
2	CKDHL0106	G:G	T:T
3	CML444	G:G	T:T
4	CML511	G:G	T:T
5	CML547	G:G	T:T
6	CML566	G:G	T:T
7	CML570	G:G	T:T
8	CML539	G:G	T:T
9	CKDHL0186	G:G	T:T
10	KS23-6 (MLN resistance donor)	T:T	C:C

T = favorable allele for SNP1 and C = favorable allele for SNP2.

### Genotyping of BC_3_F_2_ Populations for Resistance to MLN

**Figure 2:**
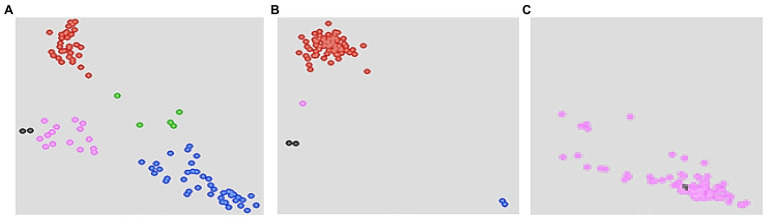
Single nucleotide polymorphism (SNP) views showing cluster plots of BC_3_F_2_ progeny and their parental lines for two SNP markers for resistance to MLN: **(A)** KASP cluster plots showing genotypes clustering for resistance (blue), susceptibility (red), and heterozygous (green) alleles. Unclassified DNA samples (purple) clustering toward the origin and closer to no treatment control (NTCs; black dots). The heterozygous genotypes (green) still segregate for MLN resistance alleles; **(B)** BC_3_F_2_ individuals lacking both SNP markers and clustered with susceptible alleles (red). Only donor parent was clustering for homozygous resistant FAM alleles (blue); **(C)** Both SNP markers were not effective hence did not discriminate between BC_3_F_2_ progeny and the parents (including donor).

Selected high quality DNA samples representing 957 BC_3_F_2_ lines selected from nine bi-parental populations and their 10 parental lines were genotyped for resistance to MLN using KASP genotyping platform at BecA-ILRI Hub Laboratory, Nairobi. The two polymorphic KASP markers mentioned earlier linked to major QTL for resistance to MLN were used (Semagn et al., 2014; Rasheed et al., 2016; Kusza et al., 2018). KASP results showed clustering of the genotypes based on the two KASP markers. Some BC_3_F_2_ and all recurrent parents clustered with susceptible homozygous HEX alleles on y-axis. The donor parent and some BC_3_F_2_ lines clustered with the resistant homozygous FAM alleles on x-axis. A few BC_3_F_2_ lines clustered for heterozygous alleles on the diagonal. However, the two markers failed to discriminate between some BC_3_F_2_ including parents (Figure 2).

**Figure 3:**
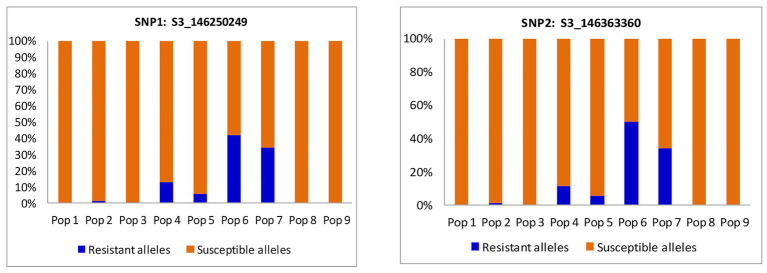
Percent distribution of BC_3_F_2_ progeny per population based on resistance and susceptibility alleles for two SNP markers linked to major quantitative trait loci (QTL) associated with resistance to MLN.

Cluster plot results indicated that 57 BC_3_F_2_ individuals were segregating for resistance to MLN. A total of 26 BC_3_F_2_ lines were homozygous (fixed) for the favorable alleles of both KASP1 (*S3_146250249*) and KASP2 (*S3_146363360*). The remaining BC_3_F_2_ lines were fixed for one locus and heterozygous for the other (Supplementary Appendix 3). The selected 57 BC_3_F_2_ lines were subjected to artificial MLN infection for phenotypic selection under field conditions. Allele distribution for each SNP marker varied among the nine populations (Figure 3). Both markers showed higher percentages of alleles for resistance to MLN among progeny from populations six and seven. Population 6 contributed most with over 40% of the population showing favorable alleles for resistance to MLN, followed by population 7 with over 30% distribution of BC_3_F_2_ progeny containing favorable alleles for resistance to MLN for both markers. Populations 1, 2, 3, and 9 contributed the least with less than 20% each for both markers.

### Phenotypic Evaluation of BC_3_F_2_ Populations for Resistance to MLN

**Figure 4:**
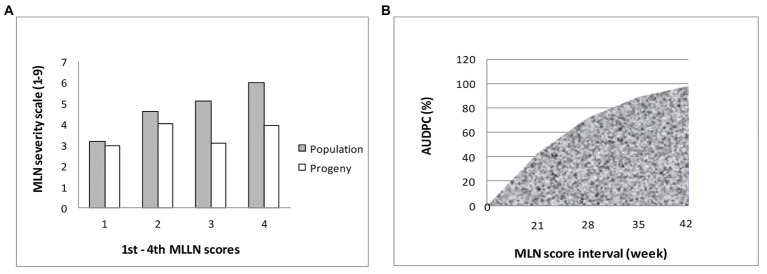
**(A)** Means of best BC_3_F_2_ progeny compared to population means across four scores; **(B)** Area under disease progress curve (AUDPC) produced by plotting AUDPC values (along y-axis) against MLN development stage (along x-axis).

The mean distribution for the populations used in the current study is presented in Table 5. The results showed that mean distribution for MLN scores ranged from 3.2 for first score to 6.0 for the last fourth score, with area under disease progress curve (AUDPC) of 133.2, respectively. A total of 19 selected BC_3_F_2_ lines including MLN resistance donor parent showed MLN severity and AUDPC below the population mean for both early and late scores. BCL02 was the most resistant line with MLN score of 3.1, which was similar to the mean of the donor parent and it had an even lower AUDPC score of 69.6 compared to the donor parent that showed a score of 81.8. When mean severity (1–9) was plotted against the score interval (in weeks), it was observed that the best performing BC_3_F_2_ lines had lower MLN mean severity across scores compared to the general population mean (Figure 4A). Similarly, the trend of the development of AUDPC showed that both disease severity and AUDPC values increased as MLN severity increased from first to fourth scores (Figure 4B). Results obtained from ANOVA are shown in Table 6. It was observed that there were variations among the BC_3_F_2_ lines for response to MLN infections under field conditions. The variability among the genotypes ranged from significant (*p* ≤ 0.05) at first MLN severity score (MLN1) to highly significant (*p* ≤ 0.01) for the fourth MLN severity score (MLN4). Similarly, the results showed highly significant (*p* ≤ 0.001) variability for AUDPC. Broad-sense heritability was detected to be very high and it ranged from of 0.84 to 0.91. Narrow-sense heritability was moderate to high with values ranging from 0.32 to 0.58, respectively.

**Table 5:** Mean scores and AUDPC for MLN severity for 10 resistant BC_3_F_2_ lines compared to donor and susceptible parents evaluated under artificial MNL infections in Naivasha for two seasons in 2018.

SN	Geno.	Pedigree	MLN1	MLN2	MLN3	MLN4	AUDPC
1	BCL02	(CKDHL0186 * 2/KS23-6):B-1019 > 1,033	2.5	2.5	2.4	3.1	69.6
2	BCL05	(CKDHL0106 * 2/KS523-5):B-1110 > 1,016	2.8	3.6	2.1	3.2	69.5
3	BCL16	(CML511 * 2/KS23-6):B-1083 > 1,008	2.8	3.6	2.5	3.2	70.3
4	BCL03	(CKDHL0106 * 2/KS523-5):B-1110 > 1,016	3.0	3.9	2.3	3.3	73.4
5	BCL18	(CML511 * 2/KS23-6):B-1083 > 1,008	2.8	4.1	3.4	3.4	82.2
6	BCL20	(CML511 * 2/KS23-6):B-1083 > 1,008	2.9	4.0	2.6	3.7	76.7
7	BCL17	(CML511 * 2/KS23-6):B-1083 > 1,008	3.0	3.7	2.8	3.9	77.8
8	BCL27	(CML511 * 2/KS23-6):B-1154 > 1,037	3.0	4.1	3.1	3.9	81.6
9	BCL31	(CML511 * 2/KS23-6):B-1154 > 1,037	2.9	4.3	2.9	4.0	82.8
10	BCL33	(CML511 * 2/KS23-6):B-1154 > 1,037	2.9	4.1	2.9	4.0	80.3
11	Check	KS23-6 (Donor parent)	2.9	4.5	3.0	3.1	81.8
12	Check	CKDHL0106 (Recurrent parent)	3.3	5.0	4.8	5.7	116.6
13	Check	CML511 (Recurrent parent)	3.6	5.1	5.5	6.6	134.5
	Mean		3.2	4.6	5.1	6.0	133.3
	LSD (0.05)		0.6	1.2	0.9	1.2	22.7
	CV%		11.8	14.6	10.3	12.1	9.9
	Min mean		1.8	2.4	2.1	3.1	69.5
	Max mean		3.9	6.5	8.1	8.8	212.7

MLN severity scores taken on a scale of 1–9, where 1–4 is resistant to moderate resistant. *MLN1*, 1st score of MLN severity taken 21 days from date of first inoculation; MLN2, 2nd score of MLN severity taken 7 days after the first score; MLN3, 3rd score of MLN severity recorded 14 days after the first score; *MNL4*, 4th score of MLN severity taken 21 days from the first score; AUDPC, area under disease progress curve calculated from the four MLN scores; LSD (0.05), Fisher’s protected least significant difference at 5% level; and CV%, coefficient of variability measures in percent.

**Table 6:** Mean squares and variance components of BC_3_F_2_ populations evaluated for two seasons for resistance to MLN in Naivasha in 2018.

Trait	Mean squares	*F*-value	*F*-prob.	*H*^2^	σ^2^_G_	σ^2^_E_
MLN1	0.4	2.25	0.024	0.84	0.92	0.17
MLN2	1.4	2.7	0.008	0.87	3.23	0.50
MLN3	1.6	4.74	<0.001	0.91	3.49	0.33
MLN4	2.1	3.09	<0.001	0.88	4.84	0.67
AUDPC	834.8	3.86	<0.001	0.90	1886.10	216.50

*MLN1*, 1st score of MLN severity taken 21 days from date of first inoculation; MLN2, 2nd score of MLN severity taken 7 days after the first score; MLN3, 3rd score of MLN severity recorded 14 days after the first score; *MNL4*, 4th score of MLN severity taken 21 days from the first score; AUDPC, area under disease progress curve calculated from the four MLN scores; H^2^_,_ broad sense heritability; σ^2^_G_, genotypic variance; and σ^2^_E_, error variance; the Degree of freedom = 61 for each trait.

**Table 7:** Some selected BC_3_F_2_ lines and a donor parent showing strong co-segregation of resistant alleles with phenotypic MLN scores under field infections.

S/N	Genotype	SNP1 (T/G)	SNP2 (C/T)	MLN1	MLN2	MLN3	MLN4
1	KS23-6 (door parent)	T:T	C:C	2.9	4.5	3.0	3.1
2	BCL5	T:T	C:C	2.8	3.6	2.1	3.2
3	Check5	T:T	C:C	1.8	2.4	3.0	3.2
4	BCL3	T:T	C:C	3.0	3.9	2.3	3.3
5	BCL10	T:T	C:C	3.0	4.4	3.6	4.2
6	BCL11	T:T	C:C	2.8	4.1	3.1	4.2

T:T and C:C indicate MLN resistant loci for SNP1 and SNP2.

### Validation of Marker Effects on Phenotypic Variations

At least 57 BC_3_F_2_ lines selected using the two KASP markers linked to major QTL for resistance to MLN were evaluated together with their parents under artificial MLN inoculation in Naivasha during first season of 2018. Phenotypic means and genotypic data were compared and co-segregations of resistance alleles with phenotype were determined (Supplementary Appendix 4). Some selected BC_3_F_2_ lines with strong allele-phenotype associations for resistance to MLN are shown in Tables 7 and 8. Additionally, 31 BC_3_F_2_ lines fixed for one of the two resistance loci showed resistant to moderately susceptible reactions to MLN with means ranging from 3.1 to 6.7. However, 20 BC_3_F_2_ lines fixed for both resistance loci, showed susceptibility to MLN with mean severity of 7.5–8.8 (data not shown).

**Figure 5:**
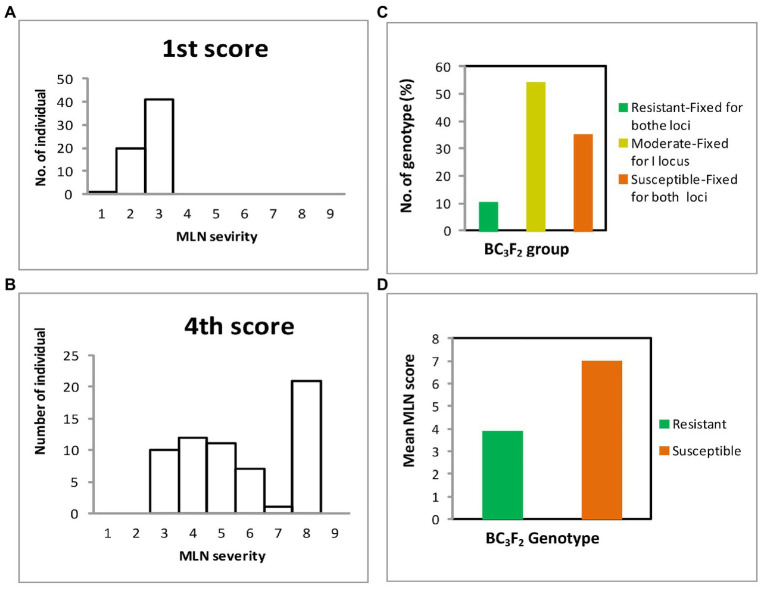
Responses of BC_3_F_2_ lines to MLN infections in the field recorded at scale of 1–9: **(A)** mean distribution of MLN severity for first score; **(B)** mean distribution of MLN severity fourth score; **(C)** mean distribution of MLN scores for resistant, moderate, and susceptible genotypes; and **(D)** mean distribution of MLN scores for resistant and susceptible groups of genotypes.

Distribution of genotypes based on their responses to MLN infection is shown in Figure 5. The recurrent parents demonstrated moderate to highly susceptible responses to MLN. Comparison between early and late scores showed that early MLN mean severity values for all genotypes were below 4.0 (Figure 5A), however, for later scores, a number of individual plants succumbed to MLN infection with disease scores above 8 (Figure 5B). Out of 57 BC_3_F_2_ lines genotyped, six were fixed for both resistance loci and showed high resistance to MLN under MLN artificial inoculation in the field, whereas 31 lines fixed for only one of the two loci demonstrated moderate resistance to the disease. Another 20 lines though fixed for both resistance loci, however, manifested high susceptibility to MLN under artificial MLN inoculation in the field (Figure 5C). Mean scores for resistant genotypes was compared to the means of the susceptible group. The resistant category demonstrated lower mean MLN score of 3.9 compared to 7.0 for the susceptible genotypes (Figure 5D). Means of the two groups were subjected to *t*-test and the results revealed highly significant differences (*p* ≤ 0.001) between means of resistant and susceptible groups of the populations (Table 9). Further, differences between variances of the two means were determined using the *F*-test and the results showed highly significant differences (*p* ≤ 0.0001) as shown below (Table 8). Consequently, 19 elite BC_3_F_2_ lines fixed for both or one locus and showing resistant to moderately resistant reaction to MLN infection were identified.

**Table 9:** Summary of *t*-test of means and *F*-test statistics for the significance of the difference between variances of means for resistance to MLN between resistant and susceptible groups of BC_3_F_2_ populations.

Df	60
Mean_(Res)_	3.9
Mean_(Sus)_	7.2
Mean_(Res)_ – mean_(Sus)_	3.35
*t*-value	−10.6
*F*-value	7.4
Prob.	0.0001

Df, degrees of freedom; *p* ≤ 0.0001 = means and variances of means are highly significantly different.

**Table 8:** List of 19 MLN resistant BC_3_F_2_ lines selected for testcross development.

SN	Pedigree	MLN1	MLN2	MLN3	MLN4	AUDPC
1	(CKDHL0186 * 2/KS23-6):B-1019 > 1,033	2.1	2.1	2.5	3.0	69.6
2	(CKDHL0106 * 2/KS523-5):B-1110 >1,016	2.1	2.1	2.0	3.0	69.5
3	(CKDHL0106 * 2/KS523-5):B-1110 > 1,016	2.1	2.1	2.0	3.0	70.3
4	(CML511 * 2/KS23-6):B-1083 > 1,008	2.1	2.1	2.5	3.0	73.4
5	(CML511 * 2/KS23-6):B-1083 > 1,008	2.1	2.7	3.0	3.0	82.2
6	(CML511 * 2/KS23-6):B-1083 > 1,008	2.1	2.2	2.5	3.5	76.7
7	(CML511 * 2/KS23-6):B-1083 > 1,008	2.1	2.7	2.5	3.5	77.8
8	(CML511 * 2/KS23-6):B-1154 > 1,037	2.1	2.2	2.5	3.5	81.6
9	(CML511 * 2/KS23-6):B-1154 > 1,037	2.1	2.7	2.5	3.5	82.8
10	(CML444 * 2/KS23-6):B-1118 > 1,008	2.6	3.6	3.5	4.0	80.3
11	(CML444 * 2/KS23-6):B-1118 > 1,008	2.1	3.1	3.0	4.0	78.9
12	(CML511 * 2/KS23-6):B-1083 > 1,008	2.1	2.2	3.0	4.0	85.4
13	(CML511 * 2/KS23-6):B-1083 > 1,008	2.1	2.2	3.0	4.0	81.6
14	(CML511 * 2/KS23-6):B-1083 > 1,008	2.1	2.2	3.0	4.0	100.1
15	(CML511 * 2/KS23-6):B-1154 > 1,037	2.1	3.2	3.0	4.0	88.6
16	(CML511 * 2/KS23-6):B-1154 > 1,037	2.1	3.2	3.0	4.0	81.4
17	(CML511 * 2/KS23-6):B-1154 > 1,037	2.1	2.6	3.0	4.0	85.2
18	(CML511 * 2/KS23-6):B-1154 > 1,037	2.1	2.7	3.0	4.0	86.5
19	(CML511 * 2/KS23-6):B-1083 > 1,008	2.1	2.2	3.0	4.5	85.2
Mean		3.17	4.60	5.09	6.03	133.28
LSD (5%)		0.40	0.74	0.55	0.73	16.87
CV %		6.80	8.17	5.98	7.18	5.81
*H*^2^		0.94	0.95	0.99	0.98	0.97
*h*^2^		0.32	0.39	0.45	0.52	0.58

The mean MLN scores and AUDPC were obtained under artificial MLN infections in Naivasha during 2018 cropping season. MLN1, MLN2, MLN3, and MLN4 were 1st, 2nd, 3rd, and 4th MLN severity scores.

## Discussion

Kompetitive allele specific PCR analysis showed clustering of some BC_3_F_2_ lines with the donor parent. This indicates that the lines could be fixed for favorable alleles of the two KASP markers for resistance to MLN. The high MLN severity observed at fourth score was attributed to an increase in disease severity with time. The genotypes under study revealed two categories in the field based on their responses to MLN incidence. The first category showed low MLN scores. This might imply that the two QTL were stable across different genetic backgrounds. Therefore, materials in this category were fixed for the two loci, hence were able to manifest resistance under field conditions and minimize MLN effects. The second group, though fixed for both loci, showed high susceptibility under field conditions, which could be due to false positive effects. This means the QTL were falsely selected for favorable alleles, while they were carrying susceptibility alleles. The reason could be that the KASP markers separated from the resistance genes during meiosis resulting in the markers being present but the genes are not. This outcome highlights the critical importance of confirming resistance of MLN under field conditions when molecular markers are used to select for resistance. The significant (*p* ≤ 0.01) variability observed among the genotypes for resistance to MLN, in this study implies that the genotypes responded differently to MLN infection. Further, statistical results showed significant AUDPC and with increased disease development for MLN, with maximum at the fourth score. Development of MLN within plant systems is rapid and is supported by viral movement and replication proteins produced by the pathogens (Mbega et al., 2016; Xia et al., 2016). Therefore, MLN quickly developed such that by the 42nd day (4th scores) after first inoculation, the disease had advanced and colonized most parts of the plant systems leading to expanded AUDPC.

Narrow sense heritability estimates for MLN scores were moderate to high indicating that resistance to MLN was mostly conditioned by additive gene action as opposed to non-additive inheritance. Additionally, the high heritability indicated that genotype played major roles in influencing the variability in MLN resistance among the individuals compared to the environment in which the experiment was conducted. In summary, the moderate to high narrow-sense heritability estimates imply the ease of transfer of the target trait from parent to offspring. High heritability estimates for disease resistance have been reported in maize (Gowda et al., 2015; Sukruth et al., 2015; Cao et al., 2017). Gowda et al. (2018) reported moderate to high heritability estimates of 0.34–0.89 for early and late MLN scores. Beyene et al. (2017) observed broad sense heritability of 69–73% for MLN resistance. The moderate to high narrow-sense heritability estimates observed in the present study also indicates that genotypes contributed significantly to the phenotypic variation observed. Therefore, identification of these lines for resistance to MLN is possible through field evaluation of the genotypes similar to findings of Pereira et al. (2015).

The present study validated two KASP markers (*S3_146250249* and *S3_146363360*) on chromosome 3, which have been reported to habor a hot spot region for various genes responsible for resistance to diseases of economic importance in maize (Gowda et al., 2015; Lohithaswa et al., 2015). *T*-test analysis revealed that means of resistant and susceptible groups were highly significant (*p* ≤ 0.0001) meaning that resistant and susceptible genotypes performed differently under MLN infections. Similarly, *F*-tests showed that differences between variances of the means were highly significant (*p* ≤ 0.0001). The findings implied that phenotypic resistance demonstrated by genotypes was highly related to the favorable alleles associated with major QTL for resistance to MLN. Consequently, 26 BC_3_F_2_ lines containing both KASP markers demonstrated resistance to MLN infections, suggesting that the two QTL were associated with phenotypic resistance in those populations. Tanweer et al. (2015) used marker assisted backcrossing for introgression of two blast resistance genes (*Pi-b* and *Pi-kh*) into a locally adapted rice line. Evaluation for blast resistance under field conditions revealed that the improved lines had higher resistance against pathotype P7.2. However, a QTL may be transferred into recipient background yet its effect may not show due to interactions (epitasis and linkage) with other genes in the new backgrounds (Hospital, 2005; Collard and Mackill, 2008). In the current stury, 20 BC_3_F_2_ lines were fixed for both KASP markers but were susceptible to MLN under field conditions, which could mainly be due to weak associations (in terms of genetic distance) between the KASP markers and target gene. This could indicate the possibility of false-positive detection of QTL. The susceptible genotypes could be because of separation of these markers with the gene. This probably indicates weak linkage between the markers and the resistance genes. We recommend the identification of more closely linked loci between these markers and the resistance genes.

## Conclusion

The current study confirmed presence of two KASP markers (*S3_146250249* and *S3_146363360*) with major effects for resistance to MLN under field conditions. Both QTL are located on chromosome 3 at a distance of 113,111 nucleotides apart. These two QTL were reproducible under different genetic and environmental conditions. The validation study confirmed that 19 superior BC_3_F_2_ lines were fixed for favorable alleles of the two QTL, and showed higher levels of resistance to MLN under artificial field inoculations. These elite BC_3_F_2_ lines represent useful parents for developing maize hybrids with resistance to MLN. Furthermore, the validated QTL can be utilized to speed up marker assisted breeding for resistance to MLN. The study identified 20 lines fixed for two KASP markers for resistance to MLN but with susceptible reaction under artificial MLN inoculations suggesting weak marker-gene linkage. We recommend the identification of additional loci between these markers and the resistance genes to strengthen the linkage. The results highlight the importance of confirmation of resistance under field conditions when molecular markers are used for selection.

## Data Availability Statement

The original contributions presented in the study are included in the article/Supplementary Material, further inquiries can be directed to the corresponding authors.

## Author Contributions

LA conducted experiments, collected data, conducted data analysis, and drafted the manuscript. NY and OS coordinated lab work at BecA, supported molecular analyses, and revised the manuscript. MJ and MS supervised field experiment, coordinated the work in CIMMYT, and reviewed the manuscript. PT, ED, BI, and PM-D supervised the experiment and revised the manuscript. MG, MO, and PB revised the manuscript. All authors contributed to the article and approved the submitted version.

### Conflict of Interest

The authors declare that the research was conducted in the absence of any commercial or financial relationships that could be construed as a potential conflict of interest.

## Footnotes

^1^www.lgcgenomics.com
