# Supplementary material for: Introgression of Maize Lethal Necrosis Resistance Quantitative Trait Loci Into Susceptible Maize Populations and Validation of the Resistance Under Field Conditions in Naivasha, Kenya
Source: Front Plant Sci. 2021 May 3;12:649308. doi: 10.3389/fpls.2021.649308 (PMC8143050; doi:10.3389/fpls.2021.649308)
Supplement: Supplementary file 1 [file Data_Sheet_2.PDF]

Supplementary Materials

Supplementary Table 1 List of SNP markers developed by CIMMYT for resistance to maize lethal necrosis

SN	SNP ID	Intertek ID	Owner	Status	Trait	Gene	Chr.	Sequence info
1	S3_44062810	snpZm0036	CIMMYT	In use	MLN	N/A	3	GTTCTTGGGCTCCGAGGATTAACGACGGGAAGGTGGCTGCAGGCCGTGCT[C/T]CCGGCAATAAGGCGGATCTGTTCTATTATCGACCCATTCCCATGGGGCGG
2	S3_144088367	snpZm0044	CIMMYT	In use	MLN	N/A	3	TTTGATGGTGCAACCTCAGCTCTTATAAACTATCTAGTTTAACATCCATAAATAAAGCAC[T/G]TATTCATGAGTATTGAACACAAGAAACAATGCTGCTAAGTTCTATTGTGTATAGATTATA
3	S3_146026612	snpZm0045	CIMMYT	In use	MLN	N/A	3	TGCTCCAGGCTCCTTGCTCTGGCAGCGTTCAAGGTAGCACTGCAGGATGGCAAGCTTCGC[A/G]GTCTCAAAGGCACAGTAGCCTAGCGCCGCAAAGCACGCGCTGTGCAGCACCCTGGGCCCG
4	S3_146966676	snpZm0046	CIMMYT	In use	MLN	N/A	3	GGGCAGCTGCACGGCGCACGCGGCGGCGTCTCGCGGCGCCCGCGTCCTGCTGCTGGAGCG[T/C]TTCGACCTGCTCCACCCGCTGGGCTCCTCGCACGGCGACTCCCGCATCATCCGGGACGCC
5	S3_133048570	snpZm0050	CIMMYT	In use	MLN	N/A	3	CGCTGCGATCCCCTCCGCCGTGGCGTCGGCGACTCCCTCTCCCGCGACGC[C/T]CTTTCATCGCTGCAGTTGATACGCCACCCGAGCGGTTCCTTCCCTCCTCT
6	S3_146250249	snpZm0052	CIMMYT	In use	MLN	N/A	3	CCGACGCGTACCGGCACCGGCGCCGCCATGGCTCTACCCATCCGCCTGCT[T/G]TCGTGGCTCGCGCTCGCCACGCTTCTCTCCGTGCCAGGTGCTGCCGCGCA
7	S3_146363360	snpZm0053	CIMMYT	In use	MLN	N/A	3	GCGTTCTGTACTGCACATTTTTAAAGGAAACCAGGACAGGTATCTAACGC[C/T]CGTCATTGTTGTGCTCAGACCTGGTACGTTTTGCAGCTACTCGAGTCATT
8	S3_146602134	snpZm0055	CIMMYT	In use	MLN	N/A	3	CGTGATGCTTCACAGGATGTTGCACTCGAGCGGAAGAGTCTGTGTCGTGC[T/C]TTTCTAGGACATGTCATGCGACGTAATGGTAGAACACTCACATGTACCTA
9	S6_6184299	snpZm0058	CIMMYT	In use	MLN	N/A	6	CGCCTGGACCTCAGCGGAAACCTCCTAGGAGGTCCAGGCGTCACCATACC[G/T]GGCTTCCTCGGCTCCCTCAGCAGCTTGGTGTACCTCAACCTCTCCGCCAT
10	S6_21007530	snpZm0061	CIMMYT	In use	MLN	N/A	6	CTATCTTGCAGCAGCATCCTACAGGACTGCAGCAAAAATCACAGCCGATC[G/A]GGCAATTTTTGTTAGACTATTGTATTAGGCTTTAGGCCCGGCTGGTCCTA
11	S6_86345596	snpZm0062	CIMMYT	In use	MLN	N/A	6	TCGATGAGGACGATCTCGTTGAGCGTGGCCGCCATCCTCCCTATCGACGG[G/C]GGGATGCAGCCGCCGAGGCGGTTGTGCGCGAGCACGAGCACGGAGGCCGG
12	S6_91855195	snpZm0063	CIMMYT	In use	MLN	N/A	6	CTCTCTGGTAGTTCTTGCCCTCGAGAACATCCGGCGCTGCGGTCTTCGCC[G/T]GTCTCGTCGTCTTCCACCAACACTGCAAAATGGCGAGCAGCAGTATTGCC
13	S6_87406549	snpZm0065	CIMMYT	In use	MLN	N/A	6	GTATCTGCGGTCATCAACTCCAGCAGCAACAGTAGTTATCCATGGGCTGAAAGACACCAG[G/T]GTTTTTGGAAATGGTCCACCATTTCCTGCAGCTTGGGCAACAAATACCCCAGCTTTCACA

14	S6_89823772	snpZm0066	CIMMYT	In use	MLN	N/A	6	TAAAATAAAGAAGCGCAGCGTCTGGGCTGGGCTCAGCGAGGGGAACGTCTTAACAGGCCG[G/C]GTTGCTAGGCCTGCGCAGCGTGCGGAGGAGGTGCAGCTGCGCCTGGGCCCGTCTGCTCCC
15	S6_157568432	snpZm0068	CIMMYT	In use	MLN	N/A	6	GCTGCAAATAAGGAGGTAGAGGCACGGCATGTTATGATCCATGTTGTCCCTCCGTAGGAA[C/A]CCTTGTCTCATTTTATTTCTATGCTGCAGGCTGAAAAGGAGGCCTTTGAAGGAGCTGAAA
16	S6_149251173	N/A	CIMMYT	In use	MLN	N/A	6	CAAGACCAACACTTTTCTTTACCATGCTTTTGCTAGGTAGGTGTCCTTGTAGCAACTGTC[A/C]AGACATTTATTTCCTTGACAGATGGATTATGTTGATAGATATTTGTAAGAACTACTATAT
17	S6_157943848	N/A	CIMMYT	In use	MLN	N/A	6	GCTCCGCCCGACCCCAGGGCTCGGACTCGGGCTAAGACCCGGAAGACGGCGAACTCCGCT[C/A]CGCCCGACCCCAGGGCTCGGACTCGGGCTAAGACCCGGAAGACGGCGAACTCCGCTCCGC
18	S6_161663671	N/A	CIMMYT	In use	MLN	N/A	6	CTGGTAAGCCGCAGAACTTGCTTCTGCCTTATCCCTGTTTCTGCGTCTCAATCTGATTAT[T/A]TGGGTTTTCCATTTGTTCTCAAGGGTTGCGGGGTTACAGGTCAGCCTTGCCTTCGGTCCC
19	S6_164081101	N/A	CIMMYT	In use	MLN	N/A	6	CCTCGTACCTGAGACGACGGTGCCTCCAGTCCCCGACGTGACTATGTCGCTCCTCGTCGA[C/A]CAAAAGGTACTGACTGATTCCCATCTTACGTCATTTCGACTCGGCCTCAACCCGCCTAGC
20	S6_164999578	N/A	CIMMYT	In use	MLN	N/A	6	TATCCTCAGACCCCAGTTCTGTCGTCGGCTGAGTTATACATGTTAGTCCACCCATATGGA[A/G]AGTTGACAAATTACTGGAGGATGCTGATGTCAAATTTGGACTGTAAGCTCGAAGTACACC
21	S6_167527305	N/A	CIMMYT	In use	MLN	N/A	6	AAATCTCTTTTGTTGTCTCTCTTGTCAAAAGTTGGCTTCTTGTGGGGAGAAGTATTGATT[T/A]TGGGAAATAGGGGGAGTTTTTGGAATCTTGAATCAATTTTCTTTGGAAAACCTCTCTTTA

Supplementary Appendix 1 List of maize parent lines used for development of bi-parental backcross populations for resistance to maize lethal necrosis in CIMMYT, Kenya during 2015-2017 cropping seasons.

Line	Name	Pedigree	MLN status	HG	MG	Adaptation
1	KS23-6	N/A	Resistant	N/A	N/A	N/A
2	CML442	(m37W/Zm607-#-B-F37SR-2-3SR-6-2-X)-8-2-X-1-B	Susceptible	A-Tester	Intermediate	Africa MA/ST
3	CML444	P43-C9-1-1-1-1-1-B	Susceptible	B-Tester	Late	Africa MA/ST
4	CML511	(CML389/ CML176)-B-29-2-2-1-B	Susceptible	B	Early/Intermediate	Africa MA/ST
5	CML537	MAS(CML206/ CML312)-23-2-1-1-B	Susceptible	A	Intermediate	Africa MA/ST
6	CML539	MAS(MSR/ CML312)-117-2-2-1-B	Susceptible	A	Early/Intermediate	Africa MA/ST
7	CML540	INTA-F2-192-2-1-1-1-B	Susceptible	A	Early	Africa MA/ST
8	CML547	DRB-F2-60-1-1-1-B	Susceptible	B	Intermediate	Africa MA/ST
9	CML548	Zm523A-16-2-1-1-B	Susceptible	A	Intermediate	Africa MA/ST
10	CML566	(LAPOSTASEQ-C7-F96-1-2-1-1-B*3/ CML444// CML444)-DH16-B	Susceptible	B	Late	Africa MA/ST
11	CML567	(LAPOSTASEQ-C7-F71-1-2-1-2-B*3/ CML539// CML539)-DH3-B	Susceptible	A	medium/Interm.	Africa MA/ST
12	CML568	(LAPOSTASEQ-C7-F71-1-2-1-2-B*3/ CML539// CML539)-DH20-B	Susceptible	A	medium/Interm.	Africa MA/ST
13	CML569	(LAPOSTASEQ-C7-F71-1-2-1-2-B*3/ CML395// CML395)-DH21-B	Susceptible	B	medium/Interm	Africa MA/ST
14	CML570	(LAPOSTASEQ-C7-F71-1-2-1-2-B*3/ CML444// CML444)-DH49-B	Susceptible	B	Late	Africa MA/ST
15	CML572	(INTA-2-1-3/INTA-60-1-2)-X-11-6-3-B*4	Susceptible	A	Early-medium	Africa MA/ST
16	CKL05017	N/A	Susceptible	A	N/A	Africa MA/ST
17	CKL05019	N/A	Susceptible	A	N/A	Africa MA/ST
18	CKDHL0106	N/A	Susceptible	N/A	N/A	Africa MA/ST
19	CKDHL0186	N/A	Susceptible	N/A	N/A	Africa MA/ST
20	CKDHL0323	N/A	Susceptible	N/A	N/A	Africa MA/ST

MA/ST= mid altitude subtropical; HG= Heterotic group; MG= maturity group

Supplementary Appendix 2 List of 21 SNP markers developed by CIMMYT for resistance to maize lethal necrosis

SN	SNP ID	Intertek ID	Owner	Status	Trait	Gene	Chr	Sequence info
1	S3_44062810	snpZm0036	CIMMYT	In use	MLN	N/A	3	GTTCTTGGGCTCCGAGGATTAACGACGGGAAGGTGGCTGCAGGCCGTGCT[C/T]CCGGCAATAAGGCGGATCTGTTCTATTATCGACCCATTCCCATGGGGCGG
2	S3_144088367	snpZm0044	CIMMYT	In use	MLN	N/A	3	TTTGATGGTGCAACCTCAGCTCTTATAAACTATCTAGTTTAACATCCATAAATAAAGCAC[T/G]TATTCATGAGTATTGAACACAAGAAACAATGCTGCTAAGTTCTATTGTGTATAGATTATA
3	S3_146026612	snpZm0045	CIMMYT	In use	MLN	N/A	3	TGCTCCAGGCTCCTTGCTCTGGCAGCGTTCAAGGTAGCACTGCAGGATGGCAAGCTTCGC[A/G]GTCTCAAAGGCACAGTAGCCTAGCGCCGCAAAGCACGCGCTGTGCAGCACCCTGGGCCCG
4	S3_146966676	snpZm0046	CIMMYT	In use	MLN	N/A	3	GGGCAGCTGCACGGCGCACGCGGCGGCGTCTCGCGGCGCCCGCGTCCTGCTGCTGGAGCG[T/C]TTCGACCTGCTCCACCCGCTGGGCTCCTCGCACGGCGACTCCCGCATCATCCGGGACGCC
5	S3_133048570	snpZm0050	CIMMYT	In use	MLN	N/A	3	CGCTGCGATCCCCTCCGCCGTGGCGTCGGCGACTCCCTCTCCCGCGACGC[C/T]CTTTCATCGCTGCAGTTGATACGCCACCCGAGCGGTTCCTTCCCTCCTCT
6	S3_146250249	snpZm0052	CIMMYT	In use	MLN	N/A	3	CCGACGCGTACCGGCACCGGCGCCGCCATGGCTCTACCCATCCGCCTGCT[T/G]TCGTGGCTCGCGCTCGCCACGCTTCTCTCCGTGCCAGGTGCTGCCGCGCA
7	S3_146363360	snpZm0053	CIMMYT	In use	MLN	N/A	3	GCGTTCTGTACTGCACATTTTTAAAGGAAACCAGGACAGGTATCTAACGC[C/T]CGTCATTGTTGTGCTCAGACCTGGTACGTTTTGCAGCTACTCGAGTCATT
8	S3_146602134	snpZm0055	CIMMYT	In use	MLN	N/A	3	CGTGATGCTTCACAGGATGTTGCACTCGAGCGGAAGAGTCTGTGTCGTGC[T/C]TTTCTAGGACATGTCATGCGACGTAATGGTAGAACACTCACATGTACCTA
9	S6_6184299	snpZm0058	CIMMYT	In use	MLN	N/A	6	CGCCTGGACCTCAGCGGAAACCTCCTAGGAGGTCCAGGCGTCACCATACC[G/T]GGCTTCCTCGGCTCCCTCAGCAGCTTGGTGTACCTCAACCTCTCCGCCAT
10	S6_21007530	snpZm0061	CIMMYT	In use	MLN	N/A	6	CTATCTTGCAGCAGCATCCTACAGGACTGCAGCAAAAATCACAGCCGATC[G/A]GGCAATTTTTGTTAGACTATTGTATTAGGCTTTAGGCCCGGCTGGTCCTA
11	S6_86345596	snpZm0062	CIMMYT	In use	MLN	N/A	6	TCGATGAGGACGATCTCGTTGAGCGTGGCCGCCATCCTCCCTATCGACGG[G/C]GGGATGCAGCCGCCGAGGCGGTTGTGCGCGAGCACGAGCACGGAGGCCGG
12	S6_91855195	snpZm0063	CIMMYT	In use	MLN	N/A	6	CTCTCTGGTAGTTCTTGCCCTCGAGAACATCCGGCGCTGCGGTCTTCGCC[G/T]GTCTCGTCGTCTTCCACCAACACTGCAAAATGGCGAGCAGCAGTATTGCC
13	S6_87406549	snpZm0065	CIMMYT	In use	MLN	N/A	6	GTATCTGCGGTCATCAACTCCAGCAGCAACAGTAGTTATCCATGGGCTGAAAGACACCAG[G/T]GTTTTTGGAAATGGTCCACCATTTCCTGCAGCTTGGGCAACAAATACCCCAGCTTTCACA
14	S6_89823772	snpZm0066	CIMMYT	In use	MLN	N/A	6	TAAAATAAAGAAGCGCAGCGTCTGGGCTGGGCTCAGCGAGGGGAACGTCTTAACAGGCCG[G/C]GTTGCTAGGCCTGCGCAGCGTGCGGAGGAGGTGCAGCTGCGCCTGGGCCCGTCTGCTCCC
15	S6_157568432	snpZm0068	CIMMYT	In use	MLN	N/A	6	GCTGCAAATAAGGAGGTAGAGGCACGGCATGTTATGATCCATGTTGTCCCTCCGTAGGAA[C/A]CCTTGTCTCATTTTATTTCTATGCTGCAGGCTGAAAAGGAGGCCTTTGAAGGAGCTGAAA
16	S6_149251173	N/A	CIMMYT	In use	MLN	N/A	6	CAAGACCAACACTTTTCTTTACCATGCTTTTGCTAGGTAGGTGTCCTTGTAGCAACTGTC[A/C]AGACATTTATTTCCTTGACAGATGGATTATGTTGATAGATATTTGTAAGAACTACTATAT
17	S6_157943848	N/A	CIMMYT	In use	MLN	N/A	6	GCTCCGCCCGACCCCAGGGCTCGGACTCGGGCTAAGACCCGGAAGACGGCGAACTCCGCT[C/A]CGCCCGACCCCAGGGCTCGGACTCGGGCTAAGACCCGGAAGACGGCGAACTCCGCTCCGC
18	S6_161663671	N/A	CIMMYT	In use	MLN	N/A	6	CTGGTAAGCCGCAGAACTTGCTTCTGCCTTATCCCTGTTTCTGCGTCTCAATCTGATTAT[T/A]TGGGTTTTCCATTTGTTCTCAAGGGTTGCGGGGTTACAGGTCAGCCTTGCCTTCGGTCCC
19	S6_164081101	N/A	CIMMYT	In use	MLN	N/A	6	CCTCGTACCTGAGACGACGGTGCCTCCAGTCCCCGACGTGACTATGTCGCTCCTCGTCGA[C/A]CAAAAGGTACTGACTGATTCCCATCTTACGTCATTTCGACTCGGCCTCAACCCGCCTAGC
20	S6_164999578	N/A	CIMMYT	In use	MLN	N/A	6	TATCCTCAGACCCCAGTTCTGTCGTCGGCTGAGTTATACATGTTAGTCCACCCATATGGA[A/G]AGTTGACAAATTACTGGAGGATGCTGATGTCAAATTTGGACTGTAAGCTCGAAGTACACC
21	S6_167527305	N/A	CIMMYT	In use	MLN	N/A	6	AAATCTCTTTTGTTGTCTCTCTTGTCAAAAGTTGGCTTCTTGTGGGGAGAAGTATTGATT[T/A]TGGGAAATAGGGGGAGTTTTTGGAATCTTGAATCAATTTTCTTTGGAAAACCTCTCTTTA

Supplementary Appendix 3 List of 21 KASP primers designed in BecA-ILRI Hub laboratory for molecular analysis of resistance to maize lethal necrosis

Primer No.	KASP Code	Primer Name	Primer Sequence	Remarks
	B0051_FAm	S3_44062810_FAm	*gaaggtgaccaagttcatgctATCCGCCTTATTGCCGGg*	Polymorphic
1	B0051_HEX	S3_44062810_HEX	*gaaggtcggagtcaacggattATCCGCCTTATTGCCGGa*	
	B0051_COm	S3_44062810_COm	AGGATTAACGACGGGAAGGT	
	B0052_FAm	S3_144088367_FAm	*gaaggtgaccaagttcatgctGTTTAACATCCATAAATAAAGCACt*	monomorphic
2	B0052_HEX	S3_144088367_HEX	*gaaggtcggagtcaacggattGTTTAACATCCATAAATAAAGCACg*	
	B0052_COm	S3_144088367_COm	AGCAGCATTGTTTCTTGTGTTC	
	B0053_FAm	S3_146026612_FAm	*gaaggtgaccaagttcatgctGGCTACTGTGCCTTTGAGACt*	
3	B0053_HEX	S3_146026612_HEX	*gaaggtcggagtcaacggattGGCTACTGTGCCTTTGAGACc*	
	B0053_COm	S3_146026612_COm	AAGGTAGCACTGCAGGATGG	
	B0054_FAm	S3_146966676_FAm	*gaaggtgaccaagttcatgctGTCCTGCTGCTGGAGCGt*	Polymorphic
4	B0054_HEX	S3_146966676_HEX	*gaaggtcggagtcaacggattGTCCTGCTGCTGGAGCGc*	
	B0054_COm	S3_146966676_COm	GTAGGCGTCCCGGATGAT	
	B0055_FAm	S3_133048570_FAm	*gaaggtgaccaagttcatgctCAACTGCAGCGATGAAAGg*	
5	B0005_HEX	S3_133048570_HEX	*gaaggtcggagtcaacggattCAACTGCAGCGATGAAAGa*	
	B0055_COm	S3_133048570_COm	GACTCCCTCTCCCGCGAC	
	B0056_FAm	S3_146250249_FAm	*gaaggtgaccaagttcatgctCTACCCATCCGCCTGCTt*	Polymorphic
6	B0056_HEX	S3_146250249_HEX	*gaaggtcggagtcaacggattCTACCCATCCGCCTGCTg*	
	B0056_COm	S3_146250249_COm	CACCTGGCACGGAGAGAAG	
	B0057_FAm	S3_146363360_FAm	*gaaggtgaccaagttcatgctACCAGGACAGGTATCTAACGCc*	Polymorphic
7	B0057_HEX	S3_146363360_HEX	*gaaggtcggagtcaacggattACCAGGACAGGTATCTAACGCt*	
	B0057_COm	S3_146363360_COm	CGTACCAGGTCTGAGCACAA	
	B0058_FAm	S3_146602134_FAm	*gaaggtgaccaagttcatgctGGAAGAGTCTGTGTCGTGCt*	monomorphic
8	B0058_HEX	S3_146602134_HEX	*gaaggtcggagtcaacggattGGAAGAGTCTGTGTCGTGCc*	

	B0058_COm	S3_146602134_COm	CTACCATTACGTCGCATGACA	
	B0059_FAm	S6_6184299_FAm	*gaaggtgaccaagttcatgctGTCCAGGCGTCACCATACCg*	monomorphic
9	B0059_HEX	S6_6184299_HEX	*gaaggtcggagtcaacggattGTCCAGGCGTCACCATACCt*	
	B0059_COm	S6_6184299_COm	TGAGGTACACCAAGCTGCTG	
	B0060_FAm	S6_21007530_FAm	*gaaggtgaccaagttcatgctGCAAAAATCACAGCCGATCg*	Polymorphic
10	B0060_HEX	S6_21007530_HEX	*gaaggtcggagtcaacggattGCAAAAATCACAGCCGATCa*	
	B0060_COm	S6_21007530_COm	CCGGGCCTAAAGCCTAATAC	
	B0061_FAm	S6_86345596_FAm	*gaaggtgaccaagttcatgctATCCTCCCTATCGACGGg*	monomorphic
11	B0061_HEX	S6_86345596_HEX	*gaaggtcggagtcaacggattATCCTCCCTATCGACGGc*	
	B0061_COm	S6_86345596_COm	CCATCCTCCTCAACTCCAAC	
	B0062_FAm	S6_91855195_FAm	*gaaggtgaccaagttcatgctGGTGGAAGACGACGAGACc*	monomorphic
12	B0062_HEX	S6_91855195_HEX	*gaaggtcggagtcaacggattGGTGGAAGACGACGAGACa*	
	B0062_COm	S6_91855195_COm	AGTTCTTGCCCTCGAGAACA	
	B0063_FAm	S6_87406549_FAm	*gaaggtgaccaagttcatgctATGGGCTGAAAGACACCAGg*	monomorphic
13	B0063_HEX	S6_87406549_HEX	*gaaggtcggagtcaacggattATGGGCTGAAAGACACCAGt*	
	B0063_COm	S6_87406549_COm	CCAAGCTGCAGGAAATGGt	
	B0064_FAm	S6_89823772_FAm	*gaaggtgaccaagttcatgctGAACGTCTTAACAGGCCGg*	monomorphic
14	B0064_HEX	S6_89823772_HEX	*gaaggtcggagtcaacggattGAACGTCTTAACAGGCCGc*	
	B0064_COm	S6_89823772_COm	GATGCGTATCTCTGGCGTTC	
	B0065_FAm	S6_157568432_FAm	*gaaggtgaccaagttcatgctGCATAGAAATAAAATGAGACAAGGg*	Polymorphic
15	B0065_HEX	S6_157568432_HEX	*gaaggtcggagtcaacggattGCATAGAAATAAAATGAGACAAGGt*	
	B0065_COm	S6_157568432_COm	ATCCATGTTGTCCCTCCGTA	
	B0066_FAm	S6_149251173_FAm	*gaaggtgaccaagttcatgctGGTGTCCTTGTAGCAACTGTCa*	monomorphic
16	B0066_HEX	S6_149251173_HEX	*gaaggtcggagtcaacggattGGTGTCCTTGTAGCAACTGTCc*	
	B0066_COm	S6_149251173_COm	CAAATATCTATCAACATAATCCAt	
	B0067_FAm	S6_157943848_FAm	*gaaggtgaccaagttcatgctAGACGGCGAACTCCGCTc*	monomorphic
17	B0067_HEX	S6_157943848_HEX	*gaaggtcggagtcaacggattAGACGGCGAACTCCGCTa*	

	B0067_COm	S6_157943848_COm	CCGTCTTCCGGGTCTTAGC	
	B0068_FAm	S6_161663671_FAm	*gaaggtgaccaagttcatgctTTTCTGCGTCTCAATCTGATTATt*	monomorphic
18	B0068_HEX	S6_161663671_HEX	*gaaggtcggagtcaacggattTTTCTGCGTCTCAATCTGATTATa*	
	B0068_COm	S6_161663671_COm	CGCAACCCTTGAGAACAAAT	
	B0069_FAm	S6_164081101_FAm	*gaaggtgaccaagttcatgctCTATGTCGCTCCTCGTCGAc*	monomorphic
19	B0069_HEX	S6_164081101_HEX	*gaaggtcggagtcaacggattCTATGTCGCTCCTCGTCGAa*	
	B0069_COm	S6_164081101_COm	CGAAATGACGTAAGATGGGAA	
	B0070_FAm	S6_164999578_FAm	*gaaggtgaccaagttcatgctTGTTAGTCCACCCATATGGAa*	monomorphic
20	B0070_HEX	S6_164999578_HEX	*gaaggtcggagtcaacggattTGTTAGTCCACCCATATGGAg*	
	B0070_COm	S6_164999578_COm	TTCGAGCTTACAGTCCAAATTt	
	B0071_FAm	S6_167527305_FAm	*gaaggtgaccaagttcatgctTCTTGTGGGGAGAAGTATTGATTt*	monomorphic
21	B0071_HEX	S6_167527305_HEX	*gaaggtcggagtcaacggattTCTTGTGGGGAGAAGTATTGATTa*	
	B0071_COm	S6_167527305_COm	AAAACTCCCCCTATTTCCCA	

Supplementary Appendix 4 Genotypic and phenotypic mean scores for 56 BC_3F_2 populations and 6 parental lines indicating co-segregation of two SNP markers associated with resistance to MLN severity scores and AUDPC recorded under artificial MLN infections in Naivasha during first season 2018.

Entry	Genotype	Pedigree	SNP1 (T/G)	SNP2 (C/T)	MLN1	MLN2	MLN3	MLN4	AUDPC
1	BCL1	(CKDHL0186*2/KS23-6):B-1019>1033	T:T	T:T	2.8	4.0	4.0	5.4	102.4
2	BCL2	(CKDHL0186*2/KS23-6):B-1019>1033	T:T	T:T	2.5	2.5	2.4	3.1	69.6
3	BCL3	(CKDHL0106*2/KS523-5):B-1110>1016	T:T	C:C	3.0	3.9	2.3	3.3	73.4
4	BCL4	(CKDHL0106*2/KS523-5):B-1110>1016	T:T	C:C	3.3	5.5	5.6	6.1	138.8
5	BCL5	(CKDHL0106*2/KS523-5):B-1110>1016	T:T	C:C	2.8	3.6	2.1	3.2	69.5
6	BCL6	(CKDHL0106*2/KS523-5):B-1110>1016	T:T	C:C	3.1	4.5	4.8	6.2	126.5
7	BCL7	(CKDHL0106*2/KS523-5):B-1110>1040	T:G	C:T	3.2	4.3	4.6	5.6	122
8	BCL8	(CKDHL0106*2/KS523-5):B-1110>1040	T:G	C:T	3.9	6.5	7.4	8.5	186.1
9	BCL9	(CKDHL0106*2/KS523-5):B-1110>1040	T:T	C:C	3.1	4.8	6.0	7.5	144.8
10	Check1	CKDHL0106	G:G	T:T	3.3	5	4.8	5.7	116.6
11	BCL10	(CML444*2/KS23-6):B-1118>1008	T:T	C:C	3.0	4.4	3.6	4.2	100.1
12	BCL11	(CML444*2/KS23-6):B-1118>1008	T:T	C:C	2.8	4.1	3.1	4.2	88.6
13	BCL12	(CML444*2/KS23-6):B-1118>1008	T:T	C:C	3.3	4.7	4.6	5.6	121.9
14	Check2	CML444	G:G	T:T	3.2	4.5	4.1	5.0	114.7
15	BCL13	(CML511*2/KS23-6):B-1083>1008	T:G	C:C	3.1	4.6	5.1	6.1	115.9
16	BCL14	(CML511*2/KS23-6):B-1083>1008	G:G	C:C	2.8	3.6	2.5	3.2	70.3
17	BCL15	(CML511*2/KS23-6):B-1083>1008	T:G	C:C	3.0	3.7	2.8	3.9	77.8
18	BCL16	(CML511*2/KS23-6):B-1083>1008	T:G	C:C	2.8	4.1	3.4	3.4	82.2
19	BCL17	(CML511*2/KS23-6):B-1083>1008	T:G	C:C	3.3	4.9	4.2	5.2	108.4
20	BCL18	(CML511*2/KS23-6):B-1083>1008	T:G	C:C	2.9	4.0	2.6	3.7	76.7
21	BCL19	(CML511*2/KS23-6):B-1083>1008	G:G	C:C	2.9	4.5	4.7	6.7	106.1
22	BCL20	(CML511*2/KS23-6):B-1083>1008	T:G	C:C	2.9	4.0	3.3	4.8	85.2
23	BCL21	(CML511*2/KS23-6):B-1083>1008	T:G	C:C	2.9	3.8	3.3	4.2	81.4
24	BCL22	(CML511*2/KS23-6):B-1083>1008	T:G	C:C	3.1	4.2	3.4	4.3	86.5

25	BCL23	(CML511*2/KS23-6):B-1083>1008	T:G	C:C	3.3	4.6	4.1	5.6	108.2
26	BCL24	(CML511*2/KS23-6):B-1083>1008	T:G	C:C	2.8	3.6	3.0	4.1	78.9
27	BCL25	(CML511*2/KS23-6):B-1154>1037	T:G	C:C	3.0	4.1	3.1	3.9	81.6
28	BCL26	(CML511*2/KS23-6):B-1154>1037	T:G	C:C	3.1	4.4	3.1	4.1	85.4
29	BCL27	(CML511*2/KS23-6):B-1154>1037	T:G	C:C	3.5	4.9	4.3	5.4	118.4
30	BCL28	(CML511*2/KS23-6):B-1154>1037	G:G	C:C	2.8	4.3	3.2	4.2	85.2
31	BCL29	(CML511*2/KS23-6):B-1154>1037	T:G	C:C	2.9	4.3	2.9	4.0	82.8
32	BCL30	(CML511*2/KS23-6):B-1154>1037	T:G	C:C	2.9	4.1	3.1	4.1	81.6
33	BCL31	(CML511*2/KS23-6):B-1154>1037	T:G	C:C	2.9	4.1	2.9	4.0	80.3
34	BCL32	(CML511*2/KS23-6):B-1154>1037	T:G	C:C	2.8	4.2	3.9	4.9	93
35	BCL33	(CML511*2/KS23-6):B-1154>1037	T:G	C:C	2.9	4.6	4.3	5.3	101
36	BCL34	(CML511*2/KS23-6):B-1154>1037	T:G	C:C	2.9	4.5	3.7	5.2	96.3
37	BCL35	(CML511*2/KS23-6):B-1154>1037	T:G	C:C	3.9	5.5	5.1	6.1	139.9
38	BCL36	(CML511*2/KS23-6):B-1154>1037	T:G	C:C	3.4	4.7	4.2	5.2	106.7
39	BCL37	(CML511*2/KS23-6):B-1154>1041	G:G	C:C	3.3	4.8	4.5	6.1	112.8
40	Check3	CML511	G:G	T:T	3.6	5.1	5.5	6.6	134.5
41	Check4	KS23-6	T:T	C:C	2.9	4.5	3.0	3.1	81.8
42	BCL38	(CML547*2/KS23-6):B-1028>1008	T:T	C:C	3.3	4.4	6.8	8.2	182.6
43	BCL39	(CML547*2/KS23-6):B-1028>1008	T:T	C:C	3.7	5.6	7.8	8.7	209
44	BCL40	(CML547*2/KS23-6):B-1028>1008	T:T	C:C	3.3	4.8	8.1	8.2	202.6
45	BCL41	(CML547*2/KS23-6):B-1028>1008	T:T	C:C	3.4	5.1	7.8	8.3	205.7
46	BCL42	(CML547*2/KS23-6):B-1028>1008	T:T	C:C	3.4	5.0	7.7	8.6	203.7
47	BCL43	(CML547*2/KS23-6):B-1028>1008	T:T	C:C	3.2	4.4	7.3	8.2	187.1
48	BCL44	(CML547*2/KS23-6):B-1028>1008	T:T	C:C	3.8	5.9	7.9	8.3	211.3
49	BCL45	(CML547*2/KS23-6):B-1028>1008	T:T	C:C	3.9	6.0	7.8	8.3	210.5
50	BCL46	(CML547*2/KS23-6):B-1028>1008	T:T	C:C	2.9	4.1	7.4	8.3	178.9
51	BCL47	(CML547*2/KS23-6):B-1028>1008	T:T	C:C	3.0	4.0	7.6	8.2	176.4
52	BCL48	(CML547*2/KS23-6):B-1092>1019	T:T	C:C	3.4	5.2	7.8	8.6	207.1

53	BCL49	(CML547*2/KS23-6):B-1092>1019	T:T	C:C	3.5	5.3	7.8	8.6	207.3
54	BCL50	(CML547*2/KS23-6):B-1092>1019	T:T	C:C	3.8	6.1	7.9	8.8	212.7
55	BCL51	(CML547*2/KS23-6):B-1092>1019	T:T	C:C	3.4	5.2	7.8	8.7	207.6
56	BCL52	(CML547*2/KS23-6):B-1092>1019	T:T	C:C	3.4	5.2	7.8	8.8	208.1
57	BCL53	(CML547*2/KS23-6):B-1092>1019	T:T	C:C	3.3	5.0	7.7	8.6	203.5
58	BCL54	(CML547*2/KS23-6):B-1092>1019	T:T	C:C	3.4	5.2	7.7	8.6	205.7
59	BCL55	(CML547*2/KS23-6):B-1092>1019	T:T	C:C	3.6	5.7	7.9	8.7	211.5
60	BCL56	(CML547*2/KS23-6):B-1028>1008	T:T	C:C	3.4	5.1	7.8	8.7	207.3
61	Check5	KS23-6	T:T	C:C	1.8	2.4	3.0	3.2	84.7
62	Check6	CML547	G:G	T:T	3.5	5.3	7.8	8.2	206
Mean					3.17	4.60	5.09	6.03	133.28
LSD (5%)					0.40	0.74	0.55	0.73	16.87
CV%					6.80	8.17	5.98	7.18	5.81
H^2					0.94	0.95	0.99	0.98	0.97

SNP1(T/G): T=resistant allele, *G*=susceptible allele; *SNP2 (C/T): C*=resistant allele, *T*=susceptible allele;*MLN1* = first score for MLN severity;MLN*2*= second score of MLN severity taken 7 days after the first score;MLN*3*=third score of MLN severity recorded 14 days after the first score; *mNL4*=fourth score of MLN severity taken 21 days from the first score; *AUDPC*=area under disease progress curve calculated from the four MLN scores; *LSD(0.05)*= Fisher's Protected Least Significant Difference at 5 % level; *MSe*=error mean square; *CV%*= coefficient of variability measures in percent; *min* and *max*= minimum and maximum mean values; and H^2= broad sense trait heritability calculated on entry mean basis.
